# Advance in Genomics of Rare Genetic Diseases

**DOI:** 10.3390/biom13101441

**Published:** 2023-09-25

**Authors:** Elena Sommariva, Milena Bellin, Chiara Di Resta

**Affiliations:** 1 Unit of Vascular Biology and Regenerative Therapy, Centro Cardiologico Monzino IRCCS, 20138 Milan, Italy; elena.sommariva@cardiologicomonzino.it; 2 Department of Biology, University of Padua, 35121 Padua, Italy; milena.bellin@unipd.it; 3 Veneto Institute of Molecular Medicine, 35129 Padua, Italy; 4 Department of Anatomy and Embryology, Leiden University Medical Center, 2333 ZA Leiden, The Netherlands; 5 Genomic Unit for the Diagnosis of Human Pathologies, IRCCS San Raffaele Scientific Institute, 20132 Milan, Italy; 6 Faculty of Medicine, Vita-Salute San Raffaele University, 20132 Milan, Italy

Recent technical breakthroughs in genotyping and bioinformatics techniques have greatly facilitated the translation of genomics into clinical care. In particular, advancements in genomics have significantly improved our understanding and treatment of rare genetic diseases. Rare diseases collectively affect approximately 2–6% of the population [1] and tend to be overlooked by the main fields of research and drug developers. However, rare genetic diseases often represent an important starting point for the development of key knowledge and for research on novel advanced therapies that could be then applied to more frequent diseases.

New available genetic data allow real disease prevalence to be established while defining the diseases according to their etiology and not only their phenotypes. The progressive reclassification of diseases has also helped in distinguishing pathologic subtypes according to their genetic cause, better establishing patients’ disease risk and assessing disease severity for personalized medicine and genotype-driven therapies [2]. Indeed, clear genomic characterizations are progressively improving the identification of disease mechanisms, and the identification of novel molecular targets is the first step toward new therapeutic approaches.

The present collection covers most of the key advancements in this area, with a specific focus on genomics approaches in rare disease research.

The clinical relevance of the genomic advances and their specific applications in rare diseases is described in two comprehensive reviews in this Special Issue, in which genomics is identified as a paradigm to understand the pathological and physiological function of genes, pathways, and mechanisms in neurogenetic research [3,4].

Specifically, Zaghi and collaborators focused their attention on the exploitation of NGS-based approaches for the diagnosis and molecular characterization of the heterogeneity of neurodevelopmental disorders [3], while Koks highlighted how the identification of an effective treatment for the rare neurogenetic Wolfram Syndrome (WFS) closer than ever thanks to genomic studies on a WFS mouse model [4].

An exemplificative study on this topic is presented by Shen et al., describing a case series of three patients with distal hereditary motor neuropathy with variations in the *HSPB1* gene. On top of a deep clinical phenotypic characterization, expanding the knowledge on the heterogeneity of the clinical presentations of the disease, the authors performed functional in vitro studies on the newly described variant p.V97L, defining its pathogenicity [5].

The understanding of the genetic causes of some rare diseases is poor or still lacking. To meliorate the current knowledge, SNP association studies can be performed by whole-genome or candidate gene approaches.

The first has been exploited by Emmert et al. in the South Tyrol CHRIS cohort, specifically on 110 cases affected by atrial fibrillation (AF) out of a general population of 10,509. A genome-wide association scan identified two novel loci, one around an SNP next to the *PBX1* gene, and another within the *PCCA* gene. The authors completed the study with a metabolic screening that revealed a consistently lower concentration of lysophosphatidylcholine a C20:3, indicating a possible role of metabolic alterations in the pathophysiology of AF [6].

The second possible approach was the focus of the work of Alele and colleagues, who investigated the association of SNPs in heat-shock proteins with heat tolerance in members of the Australian military with a history of exertional heat stroke. They identified a clear association between the HSPA1B genetic variant at the g.31829044 locus and heat tolerance [7]. This result could pave the way for the screening of a genetic-risk biomarker.

It is important to point out that genome-wide association approaches may not reveal a significant role of SNPs in rare diseases, such as in the isolated form of nonsyndromic cleft palate. For this, Iovino and coworkers assessed the overlap between the genes harboring ultra-rare variants, detected through whole-exome sequencing of 35 patients, and the enriched gene set implicated in the pathobiology of nonsyndromic cleft palate. They found that *COL2A1* and *GLI3* were particularly enriched in the ultra-rare variant, thus constituting candidate genes that may contribute to the individual risk of disease [8].

Rare diseases still suffer from a low genetic diagnostic yield.

An example of this is in the study by Mazzaccara et al., who described the prevalence of genetic variants in a southern Italian cohort of 133 patients with inherited cardiomyopathies and channelopathies. The relatively low yield of definitive genotyping, due to both the strength of gene–disease association and the identification of variant of unknown significance, as defined by the ClinGen consortia, reinforces the need to continue screening, at least for research purposes, uncommon genes to gain more knowledge and increase the diagnostic sensitivity of the genetic testing of inherited cardiomyopathies and channelopathies [9].

Moreover, it is important to underline that beyond the main causative pathogenic mutations, common variants can be used to better classify a disease through genotype–phenotype studies. Indeed, common variants of the genes associated with cardiomyopathies and channelopathies may better define disease prognosis and the risk stratification of arrhythmogenic cardiomyopathy patients. Interestingly, Lippi and coworkers demonstrated that 28 polymorphisms were associated with an increased risk of arrhythmic events during patient follow-up, 5 of them with increased levels of the inflammation markers, while the others were associated with the right- vs. left-dominant forms of the disease [10]. In addition, the authors identified high-impact variants of genes not previously associated with arrhythmogenic cardiomyopathy, which may have a relevant role for defining its pathogenesis and for diagnostic purposes [10].

A genotype–phenotype correlation was performed in the study by Storoni et al. in a cohort of 675 Dutch osteogenesis imperfecta patients, in which they identified that carriers of dominant negative COL1A1 or COL1A2 mis-sense pathogenic variants require less healthcare attention than the other groups [11]. The results of the study could have the potential to improve the currently used Sillence classification [12].

Improving the molecular classification often means ameliorating the clinical management of patients, as described by Peretto and collaborators. Indeed, myocardial inflammation is increasingly recognized as both a phenotype and an active modulator of the pathogenesis of genetic cardiomyopathies. The review by Peretto et al. [13] highlights the genetic basis of myocardial inflammation in nonischemic cardiomyopathies and the reported molecular mechanisms. It aims to prompt future research towards the identification of novel treatment targets for better patient management.

Finally, advances in genomics have been achieved through the discovery of different categories of noncoding RNA, such as circular RNA (CircRNA), thanks to the development of more sensitive sequencing technologies. This is the topic of the literature review by Caba and coworkers, which elucidates how CircRNAs can regulate transcription, splicing, microRNA action, and protein–protein interactions, modulate the pathogenic mechanisms of some diseases and play a potential role as biomarkers [14].

In conclusion, the present Special Issue provides an in-depth look at insights into rare diseases thanks to ever-improving advances in genomics (Figure 1).

## Figures and Tables

**Figure 1:**
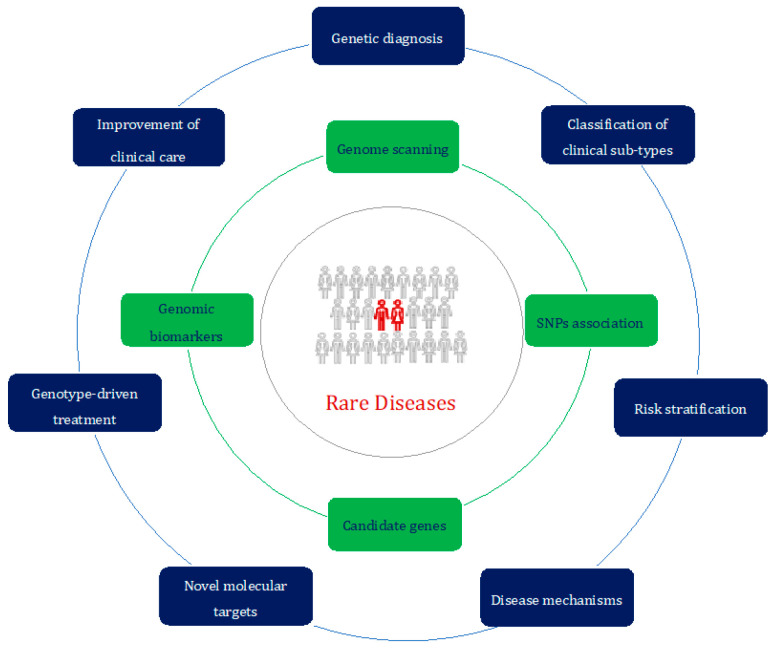
The rare-disease-centered approach of the genomic era. Different study approaches (in green) can be exploited to achieve a better characterization and subsequent management (in blue) of patients affected by rare diseases (in red).
